# Studying membrane proteins with MicroED

**DOI:** 10.1042/BST20210911

**Published:** 2022-02-22

**Authors:** Marc J. Gallenito, Tamir Gonen

**Affiliations:** 1 Department of Biological Chemistry, University of California, Los Angeles, Los Angeles CA 90095, U.S.A.; 2 Department of Physiology, University of California, Los Angeles, Los Angeles CA 90095, U.S.A.; 3 Howard Hughes Medical Institute, University of California, Los Angeles, Los Angeles CA 90095, U.S.A.

**Keywords:** cryo-EM, microcrystal electron diffraction, MicroED, transmembrane proteins

## Abstract

The structural investigation of biological macromolecules is indispensable in understanding the molecular mechanisms underlying diseases. Several structural biology techniques have been introduced to unravel the structural facets of biomolecules. Among these, the electron cryomicroscopy (cryo-EM) method microcrystal electron diffraction (MicroED) has produced atomic resolution structures of important biological and small molecules. Since its inception in 2013, MicroED established a demonstrated ability for solving structures of difficult samples using vanishingly small crystals. However, membrane proteins remain the next big frontier for MicroED. The intrinsic properties of membrane proteins necessitate improved sample handling and imaging techniques to be developed and optimized for MicroED. Here, we summarize the milestones of electron crystallography of two-dimensional crystals leading to MicroED of three-dimensional crystals. Then, we focus on four different membrane protein families and discuss representatives from each family solved by MicroED.

## Introduction

Microcrystal electron diffraction (MicroED) is a structural determination technique that integrates the two methodological pillars of structural biology — crystallography and electron microscopy (EM) [1,2]. As of the year 2021, X-ray crystallography accounts for ∼130 000 protein structures in the Protein Data Bank (PDB) out of roughly 150 000 entries. Even in its success, the production of large well-ordered crystals remains a bottleneck because, in many cases, proteins fail to crystallize or reach the crystal sizes needed for X-ray diffraction. To bypass this hurdle in X-ray crystallography, the MicroED method was developed with which extremely small three-dimensional (3D) crystals are studied by electron diffraction while in a frozen-hydrated state [1,3]. In stark advantage to X-rays, a beam of electrons interacts more strongly with matter while inflicting considerably less damage [4], allowing the use of crystals with sizes orders of magnitude smaller than X-ray crystallography normally requires.

**Figure 1.**
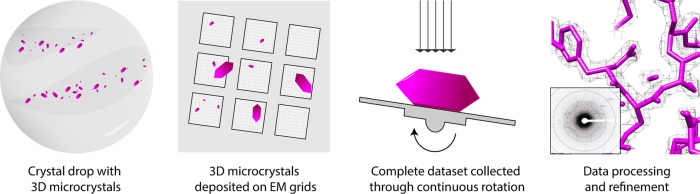
Schematic flow of the MicroED method.

For MicroED, microcrystals are prepared by standard crystallization procedures [1]. Once crystallization conditions are identified, the crystal solution is applied on to a carbon-coated EM grid [1,2,5]. Following blotting and vitrification, the grids are loaded onto a transmission electron cryo-microscope (cryo-TEM) operating in diffraction mode. Candidate crystals are identified and then used to collect a complete diffraction dataset by exposure to an ultra-low dose (<1 e^− ^Å^−2^) of electron beam while the crystal is continuously rotated. As the crystal is rotated, MicroED data are collected as a movie using a fast camera as a continuous series of frames that finely sample the reciprocal space. Because continuous rotation MicroED is analogous to the rotation method in X-ray crystallography [3] data processing and finally structure building and refinement are carried out by using existing crystallographic software [1–3,5,6] (Figure 1).

MicroED has opened possibilities for structures ranging from small molecules [7–13] to macromolecular protein assemblies [14,15] with some at an impressive atomic resolution. To name a few, protein models such as catalase and lysozyme [1,16], the toxic core of α-synuclein [17] and the amyloid core of Sup35 prion protein [18] have been solved by MicroED. Furthermore, MicroED has proven useful in the structural elucidation of challenging classes of proteins such as membrane-embedded proteins like ion channels and G protein-coupled receptors (GPCRs) [19–22]. Membrane proteins represent a key target of structural biology because of their physiological significance and importance for drug development. The amphipathic nature of membrane proteins renders them hard to express, solubilize, purify and study structurally. In recent years, an increase in reported membrane protein structures was seen because of major efforts in developing and improving structural and biochemical techniques for handling these targets.

## Membrane protein mimetic systems

Membrane proteins are a class of amphipathic proteins that reside on cell membranes where they mediate signal transduction, mediate enzymatic functions, involved in adhesion, signaling and transport of essential nutrients as well as secretion of wastes [23]. Not surprisingly, membrane proteins play major roles in human health and disease. It is known that a third of all proteins identified in the human proteome are classified as membrane proteins [24] but they disproportionally account for more than half of all drug targets [25] and yet represent but a small fraction of all structures determined to date. Therefore, understanding membrane protein function by obtaining atomic-level structural information is indispensable to drug discovery, design and optimization.

Integral membrane proteins contain one or more segments buried in the lipid bilayer flanked by hydrophilic regions exposed to either the internal or external side of the cell. These membrane-spanning segments are frequently α helices or more rarely multiple β strands that can span the phospholipid bilayer as a barrel. Interactions of amino acid residues containing hydrophobic side chains and the fatty acyl groups of the lipids facilitate the insertion of transmembrane proteins into the hydrophobic lipid membrane [26] and help maintain a hydrophobic seal. Moreover, charged residues, like arginine or lysine typically interact with lipid headgroups to create an electric seal [27]. To understand membrane proteins, it is necessary to transfer them from their native membranes to a more tractable environment. This includes extraction by a carefully selected detergent [28] followed by purification, reconstitution and crystallization.

Protein crystallization remains unpredictable and empirical and membrane proteins represent even tougher targets. The amphipatic nature of membrane proteins and their dynamic nature makes them difficult materials to form well-ordered crystals. Moreover, the use of detergents in membrane protein production adds another layer of complexity to crystal formation because crystal contacts can be hindered by large detergent micelles [29,30]. Therefore, several different detergents are typically screened in crystal assays to produce high-quality crystals and this process can be laborious and expensive.

Although detergents provide a convenient means of solubilizing and handling membrane proteins, they also come with destabilizing effects that may disrupt the membrane protein structures as well as function [31]. As an alternative, another class of solubilizing agent have been designed called amphipols [32]. In contrast with detergents, ampiphols are polymers that rigidly wrap the transmembrane regions of membrane proteins. Another class are lipid nanodiscs that consist of a belt of membrane scaffold protein derived from apolipoprotein A-1 encapsulating a circular fragment of the phospholipid bilayer. Nanodiscs closely resemble the lipid environment of cellular membranes which makes them attractive for elucidating membrane protein conformational states [33].

Other technologies allow membrane protein crystallization and structure determination directly in a lipid system. Crystallization methods such as bicelle crystallization and lipidic-cubic phase (LCP) have been extremely successful. With the bicelle method, aqueous lipid-detergent assemblies form bilayered disks [29,30,34] that surround the membrane protein of interest keeping it soluble yet inside a lipidic environment. With the LCP, a 3D matrix of densely packed lipid vesicles in a continuous bilayer engulf the membrane proteins where together they form a 3D crystal [35,36]. The success of LCP crystallization has produced over 120 structures of membrane proteins including important drug targets such as GPCRs [37]. However, regardless of the crystallization approach, membrane protein crystals tend to be small, unstable and difficult to handle because they are typically also much more fragile than crystals of soluble proteins.

## Electron crystallography of two-dimensional crystals

**Figure 2.**
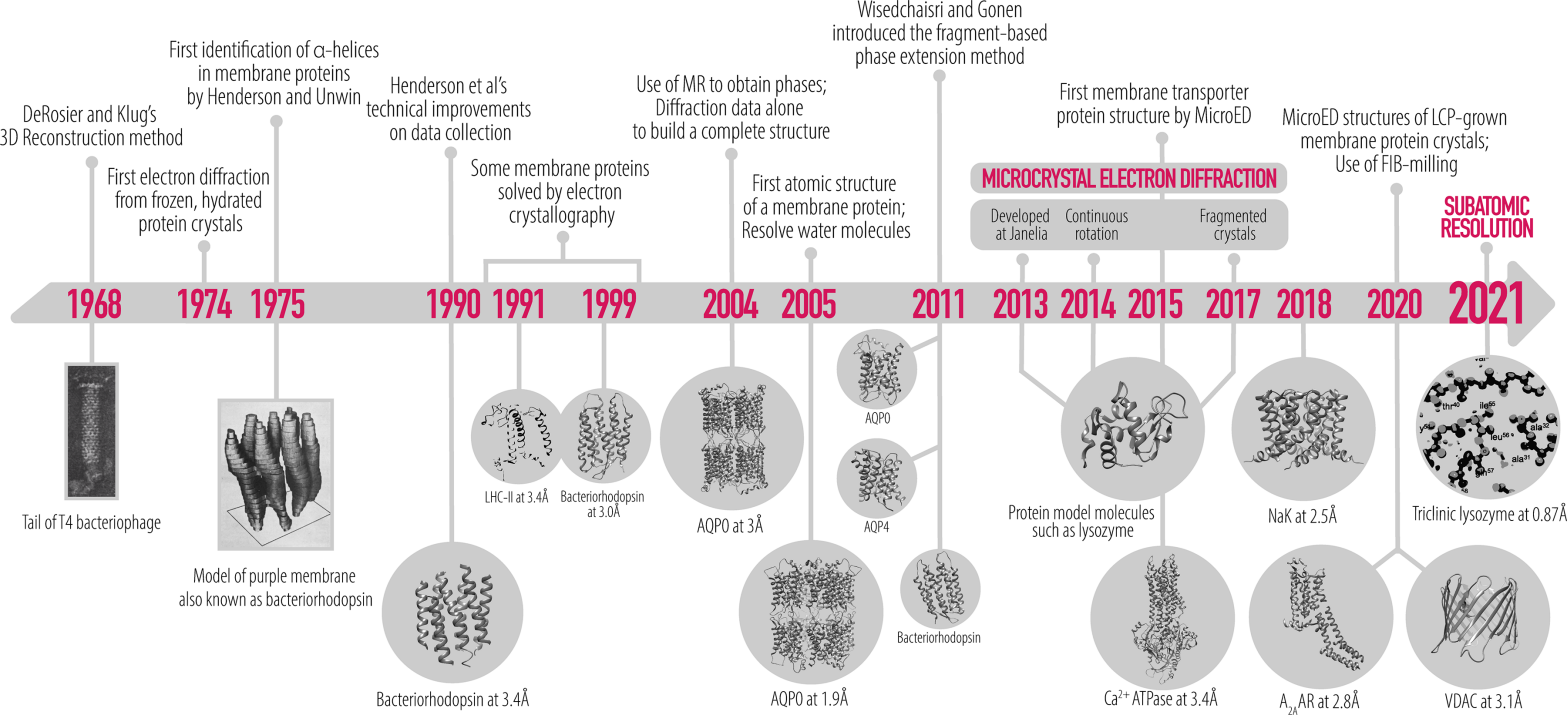
Timeline of 2D electron crystallography of membrane proteins leading to MicroED.

Some membrane proteins that are abundantly expressed in cells naturally form ordered arrays within the plasma membrane. Classic examples of naturally occurring arrays are the hexagonal lattice of bacteriorhodopsin (bR) [38] and the square arrays of aquaporins (AQP) [39] (Figure 2). In the early days of electron cryomicroscopy (cryo-EM), one of the popular techniques was that of electron crystallography of two-dimensional (2D) crystals. Much of the method was developed using naturally occurring 2D crystals of bR. Images of bR 2D crystals were recorded under cryogenic conditions at varying tilt angles and of crystals oriented in different ways on the grid support. When all projections were merged together a 7 Å resolution map of bR was calculated. This was the first structure of a membrane protein where transmembrane helices were observed. The field of electron crystallography undertook some methodological improvements that over the next decade resulted in a 3 Å structure of bR and several other membrane proteins [39–41]. In 2005, the structure of the water channel aquaporin-0 was determined by electron diffraction to 1.9 Å resolution from double-layered crystals and both lipid and protein were resolved [27]. Importantly, images, which provide phase information, were not included in the AQP0 study. Instead, the entire data set consisted only of electron diffraction data which after merging were phased by molecular replacement methods. Later, phase extension methods were described detailing additional alternative phasing methods to imaging [42]. The AQP0 study [27] was the first time that atomic resolution was obtained for proteins by cryo-EM and in which water molecules were observed.

When naturally occurring 2D arrays are not available, membrane proteins can be crystallized in membranes by slow dialysis. Membrane protein 2D crystals are produced from detergent-solubilized protein dialyzed into a lipidic environment. Slow removal of detergent molecules in the presence of detergent-solubilized lipids allows the formation of vesicles, tubules and/or sheets into which the membrane proteins insert and densely pack into 2D arrays [43]. Perfect 2D crystals are thin, flat and rigid, such was the case for AQP0. Because 3D crystals have more allowable plane groups than 2D crystals [44], it is rather challenging to induce a protein to only form 2D crystals and typically 2D sheets may stack on top of one another assembling into thin 3D crystals and these can be readily studied by MicroED.

## Membrane protein structures by MicroED

Because many membrane protein 2D crystals tend to stack and naturally pack into thin 3D crystals it became important to develop a robust method capable of extracting the necessary information for structure solution from such thin crystals. In 2013, the method MicroED was developed for exactly this purpose. Lysozyme, which is an excellent soluble protein test sample was the first protein structure solved by electron diffraction of multi-layered (about 10 layers) 3D crystals [1]. Continuous rotation MicroED improved the data collection procedure to the point where an entire data set could be collected from a single nanocrystal to atomic resolution [3]. The crystal is slowly but continuously rotated in a single direction while the diffraction data is collected using a fast camera as a movie. Because the crystal is continuously rotated during the exposure, each frame in the movie contains a wedge of reciprocal space. When at least 20° of phi is collected, indexing can be done without any au priori knowledge of the unit cell parameters and with continuous rotation, the data is processed using X-ray crystallographic software. Since 2013, several improvements to the data collection procedure, sample preparation protocols and data processing progressively improved the attainable resolution of lysozyme from 2.9 to 1.8 Å resolution [1,3,6] and with the use of direct electron detectors in counting mode was recently reported at 0.87 Å [45]. Similarly, high resolutions have been reported for several proteins by MicroED. The original MicroED work, and the technical developments that came after, makes the technique a highly viable option for the advancement of membrane protein structural biology.

**Figure 3.**
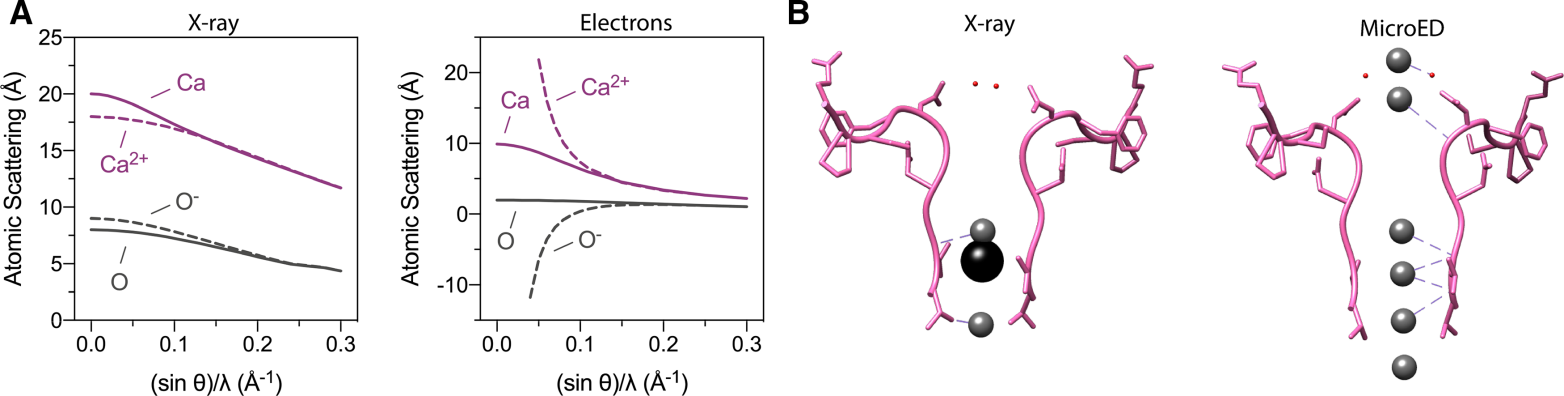
X-ray versus MicroED. (**A**) Atomic scattering values of O and Ca species by electrons and X-ray from the *International Table of Crystallography*. (**B**) NaK ion binding sites were determined by X-ray crystallography and MicroED. The Na^+^, Cs^+^ and water molecules are represented as gray, black and red sphere, respectively.

Soon after Shi et al. [19] published MicroED's proof of principle, the technique was used for membrane proteins including the Ca^2+^-ATPase and the non-selective ion channel NaK [20] which demonstrated the advantage in generating Coulomb potential maps. Electron scattering by EM is dependent on the charge distribution in the sample, since scattering occurs due to the Coulomb forces between the incident electrons and the sample molecule. In biological molecules, this would allow visualization of positively or negatively charged residues and metal ion charged states. This is fundamentally different to scattering in X-ray, where X-rays are scattered by the electron cloud of an atom, thus does not depend on the net charge of the atom (Figure 3A). Coulomb potential maps offer a breadth of electrostatic information on metal ions and charged residues that can provide insights into enzymatic function and mechanism, as was the case for Ca^2+^-ATPase and the sodium-conducting channel NaK.

Ca^2+^-ATPase is a P-type ATPase capable of pumping Ca^2+^ into the sarcoendoplasmic reticulum lumen. The detailed mechanism on the transport cycle of Ca^2+^-ATPase has been reported through biochemical and structural studies. In 2000, the first atomic resolution structure of Ca^2+^-ATPase has been solved by X-ray crystallography at 2.6 Å [46], and several other conformational states have been reported thereafter [47–49]. Before this was achieved, Ca^2+^-ATPase has eluded structural determination because it can only form tubular crystals in the absence of Ca^2+^ and 3D microcrystals in the presence of millimolar Ca^2+^ [50,51]. Both of these crystal types are not suitable for X-ray analysis and only produced low-resolution structures [52]. When MicroED was used the structure of Ca^2+^-ATPase was determined and charge properties described. MicroED coulomb potential maps revealed pronounced details of the two Ca^2+^ binding sites, including charged states of acidic residues Asp800 and Glu908 [19] (Figure 5A). Moreover, assigning charged states with less ambiguity can be extended to other membrane protein types, such as ion channels. Ion channels allow the permeation of specific solutes by creating a pore through the lipid bilayer. MicroED studies of the non-selective sodium channel NaK allowed the determination of two new conformational states of the tetrameric sodium channel [20]. One of the two was a transient state where a partially hydrated Na^+^ was bound at the entrance of the selectivity filter shedding light to how Na^+^ enters the channel (Figure 3B). This state has not been structurally captured by X-ray crystallography previously [53,54]. Because MicroED produces Coulomb potential maps, Na^+^ ions within the channel were unambiguously identified (Figure 5B).

Recently, two new studies reported the use of MicroED in LCP- and bicelle-crystallized membrane proteins [21,55]. As mentioned, crystals grown by these methods tend to be small (1–5 μm) and typically unfit for X-ray analysis yet too large for the electron beam to penetrate. However, these methods are preferred crystallization for membrane protein structure elucidation since they resonate with the natural lipid environment. Moreover, these methods use a lipid matrix with high viscosities not ideal for deposition onto EM grids, thus sample preparation for MicroED is extremely challenging for these types of samples. To circumvent this, crystallization additives and/or cryo-focused ion beam (FIB) milling may be applied to the crystals. For example, the additive 2-methyl-2,4-pentanediol (MPD) was used to reduce the viscosity of LCP-grown crystals of the GPCR human adenosine A_2A_ (A_2A_R) receptor [55]. The addition of MPD, as well as lipase, polyethyleneglycol (PEG) and jeffamine M600, converts the LCP matrix into a sponge phase, a less viscous liquid analog of cubic phase [36]. This allows better blotting of the crystal excess solution to the EM grid. Moreover, the sponge phase is thought to benefit membrane proteins with large extramembrane domains [56]. This approach had only limited success but milling using a cryo-SEM/FIB seems to be more tractable.

**Figure 4.**
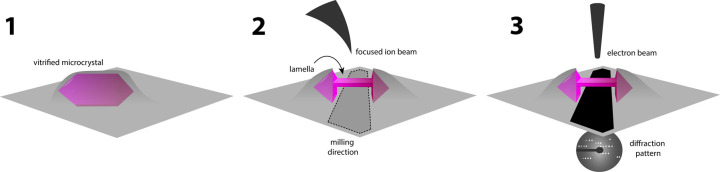
Typical workflow of cryo-FIB milling of crystals for microED.

**Figure 5.**
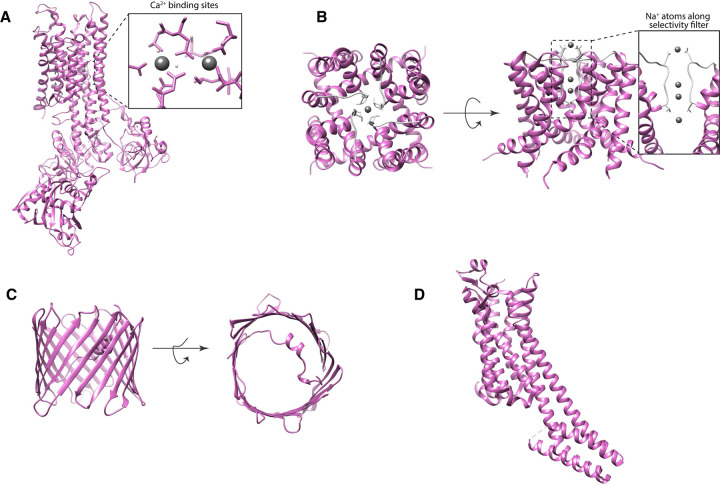
MicroED structures of membrane proteins. MicroED structures of membrane proteins: Ca^2+^-ATPase (**A**) and NaK (**B**) showing (in boxes) resolved ion species within the molecule, Ca^2+^ and Na^+^ as grey spheres, and in lipids: (**C**) VDAC in bicelles and (D) A_2A_AR in LCP.

FIB milling has been more successful in thinning out membrane protein crystals, and removing thick lipid deposits around crystals before MicroED analyses. Figure 4 shows the typical workflow of crystal thinning for MicroED. Crystals are deposited on an EM grid and vitrified following standard protocol, the grid is loaded onto a cryo-FIB/SEM. Using SEM as a secondary electron imaging, target crystals can be identified. For milling, a focused beam of ions is scanned over the top and bottom layer of the crystal until proper thickness (∼200 nm) is achieved. This is directly performed on the EM grid which would allow subsequent diffraction analysis in a cryo-TEM. Crystals grown in bicelles of a mutant of the murine voltage-dependent anion channel (VDAC) produced thin plate-shaped microcrystal shards suspended in a viscous lipid matrix. Because of the thickness of the solution, several blotting conditions were performed tested to deposit the crystals onto grids. Selected crystals were then cryo-FIB milled to thin crystalline lamellae of ∼200 nm thickness and used for MicroED collection. This yielded a 3.1 Å resolution structure of a new mutant form of VDAC (Figure 5C) [21]. While several structures of VDAC in detergent and lipid matrices were already reported by X-ray crystallography [57–61], the essential VDAC K12E mutant only produced small crystals not amenable for X-ray crystallography, in stark contrast with the large crystals of its wildtype form.

More than 800 different GPCRs exist in humans and these proteins are the largest pharmaceutical targets [62,63]. Traditional crystallography, such as vapor diffusion crystallography, often do not yield crystals of GPCRs. Owing to the intricate interactions of GPCRs and lipids, lipid-based crystallization techniques can maintain GPCRs in crystallizable conformations. However, these crystals tend to be minute in sizes and very hard to handle. Several strategies were successfully used to obtain structures from these microcrystals such as rastering and the use of microfocus beamlines and X-ray-free electron lasers (xFELs) [63–65]. These strategies can be laborious and costly because of limited access to xFEL and the amount of material needed can be limiting as well. In a recent study, the adenosine A_2A_ receptor (A_2A_AR) was successfully determined by MicroED directly from nanocrystals grown in LCP. By converting the LCP matrix into the sponge phase and cryo-FIB milling the crystals into 200-nm thick lamellae, the structure of A_2A_AR was solved at 2.8 Å from a single nanocrystal [22] (Figure 5D). By comparison, the X-ray structure of this receptor was determined to 1.8 Å resolution by using many more crystals of much larger size [66,67]. Through this work, MicroED bypasses common challenges to GPCR crystallography and structural analysis and promises to enable structure determination from samples that were until recently beyond our reach.

## Concluding remarks

MicroED unlocks new strategies for membrane protein structural biology. Since 2013, four membrane protein structures have been reported by the method in which, regardless of the crystallization technique, high-resolution structures can be attained. MicroED's ability to obtain structures from tiny and single crystals abolishes limitations imposed by the intrinsic properties of membrane proteins especially those that crystallize poorly and purify in low quantities. Through Coulomb potential maps, MicroED provides unambiguous assignment of charges of ions and residues found within the protein that can provide meaningful detail into mechanisms of transporters and channels. Moreover, the combination of LCP and MicroED allows for the investigation of lipid interactions vital for membrane protein structures, carried out through the application of FIB milling. MicroED, in combination with LCP crystallography and Coulomb potential maps, prove to be a powerful technique that delivers high-resolution structures and details underlying molecular mechanisms of membrane proteins. Together with other cryo-EM modalities, MicroED promises to transform the field of structural biology.

**Perspective**

- MicroED can deliver atomic resolution structures from vanishingly small crystals. This cryo-EM method is gaining momentum and becoming an important player in determining structures of membrane proteins embedded in a lipid matrix.
- By using MicroED structures of important material can be determined and charge properties probed in a way which is not possible by X-ray crystallography.
- Now that methods for studying membrane proteins in lipids have been developed and demonstrated the path is clear for examining samples that were thus far beyond reach.

## Abbreviations

AQP: aquaporins
bR: bacteriorhodopsin
cryo-EM: electron cryomicroscopy
cryo-TEM: transmission electron cryo-microscope
EM: electron microscopy
FIB: focused ion beam
GPCRs: G protein-coupled receptors
LCP: lipidic-cubic phase
MicroED: microcrystal electron diffraction
MPD: methyl-2,4-pentanediol
VDAC: voltage-dependent anion channel
xFELs: X-ray-free electron lasers
